# Co-housing of Rift Valley Fever Virus Infected Lambs with Immunocompetent or Immunosuppressed Lambs Does Not Result in Virus Transmission

**DOI:** 10.3389/fmicb.2016.00287

**Published:** 2016-03-07

**Authors:** Paul J. Wichgers Schreur, Lucien van Keulen, Jet Kant, Nadia Oreshkova, Rob J. M. Moormann, Jeroen Kortekaas

**Affiliations:** ^1^Department of Virology, Central Veterinary Institute, Part of Wageningen University and Research CentreLelystad, Netherlands; ^2^Virology Division, Faculty of Veterinary Medicine, Department of Infectious Diseases and Immunology, Utrecht UniversityUtrecht, Netherlands

**Keywords:** Rift Valley fever virus, transmission, epidemiology, risk assessment, contact-exposure, horizontal transmission

## Abstract

Rift Valley fever virus (RVFV) is transmitted among susceptible animals by mosquito vectors. Although the virus can be isolated from nasal and oral swabs of infected animals and is known to be highly infectious when administered experimentally via oral or respiratory route, horizontal transmission of the virus is only sporadically reported in literature. We considered that immunosuppression resulting from stressful conditions in the field may increase the susceptibility to horizontally transmitted RVFV. Additionally, we reasoned that horizontal transmission may induce immune responses that could affect the susceptibility of contact-exposed animals to subsequent infection via mosquito vectors. To address these two hypotheses, viremic lambs were brought into contact with sentinel lambs. One group of sentinel lambs was treated with the immunosuppressive synthetic glucocorticosteroid dexamethasone and monitored for signs of disease and presence of virus in the blood and target organs. Another group of contact-exposed sentinel lambs remained untreated for three weeks and was subsequently challenged with RVFV. We found that none of the dexamethasone-treated contact-exposed lambs developed detectable viremia, antibody responses or significant increases in cytokine mRNA levels. Susceptibility of immunocompetent lambs to RVFV infection was not influenced by previous contact-exposure. Our results are discussed in light of previous findings.

## Introduction

Rift Valley fever virus (RVFV) causes considerable morbidity and mortality among domesticated ruminants and occasionally humans. RVF outbreaks are generally preceded by massive expansions of mosquito populations, triggered by periods of heavy rainfall. RVFV has a very broad host range, but domesticated ruminants play a key role in the epidemiology of the disease by developing sufficiently high viremia to allow transmission to susceptible mosquitoes. The virus is currently confined to the African continent, the Arabian Peninsula and several islands off the coast of Southern Africa but continues to expand its habitat. Future incursions into previously unaffected areas could have dramatic consequences as mosquito species associated with transmission of the virus in endemic areas are globally prevalent (Bird et al., 2009; Pepin et al., 2010).

The earliest and perhaps most elegant studies on RVFV transmission were performed by Daubney and Hudson, who first characterized and named the disease (Daubney et al., 1931). Subsequent studies by Findlay revealed that experimental infection can be achieved by intramuscular, intravenous, subcutaneous, intraperitoneal, intracerebral, intranasal, or conjunctival inoculation (Findlay, 1932). Importantly, inhalation of virus-containing aerosols was also found to result in infection (Brown et al., 1981; Reed et al., 2013; Hartman et al., 2014).

Whereas experimental infection via artificial inoculation routes yielded consistent results, those obtained from studies on natural exposure routes, including contact, inhalation, or ingestion seem inconsistent. Daubney found that contact-exposed lambs did not develop disease (Daubney et al., 1931), whereas Weiss reported that RVFV was able to spread from infected mice to suckling mice via handling (Weiss, 1957). In one study, mice and rats did not become infected after being fed livers and spleens from infected animals (Findlay, 1932), whereas others reported transmission among mice via cannibalism (Mims, 1956). Easterday et al. (1962) successfully infected lambs by swabbing the buccal mucosa, demonstrating that lambs can be infected with RVFV via oral route. They also found that mice, hamsters, non-human primates, and lambs can be infected via respiratory route (Easterday et al., 1962; Easterday, 1965). In more recent studies involving mice and non-human primates, efficient infection via the respiratory route was confirmed (Reed et al., 2013; Hartman et al., 2014). Several studies reported isolation of the virus from oral and nasal swabs and two studies reported horizontal transmission to a single contact-exposed sheep (Harrington et al., 1980; Busquets et al., 2010). The combined results of these previous studies suggest that RVFV can be transmitted horizontally, but also that transmission via these routes is either very inefficient or rarely results in disease. Considering that animal trials are generally performed with animals of optimal physical health, we anticipated that animals suffering from immunosuppression resulting from co-infections or stressful conditions in the field might be more susceptible to horizontally transmitted RVFV. In addition, we considered that horizontal transmission to immunocompetent animals may result in local, subclinical infections with associated innate or adaptive immune responses that could change the susceptibility to infection via mosquito bite.

In the present work, we investigated the epidemiological significance of horizontal transmission by co-housing infected lambs with dexamethasone-treated lambs and by evaluating the susceptibility of immunocompetent lambs to experimental RVFV infection following contact exposure. The results suggest that contact-exposure of lambs does not result in disease, even when animals are immunocompromised, and that contact exposure does not significantly affect the susceptibility to infection.

## Materials and Methods

### Ethics Statement

All animal experiments were conducted in accordance with the Dutch Law on Animal Experiments (Wod, ID number BWBR0003081) and approved by the Animal Ethics Committee of CVI-Lelystad. To minimize suffering of the animals during the experiment, lambs were humanely euthanized when they could no longer be stimulated to drink, feed, or stand.

### Preparation of the challenge virus

Rift Valley fever virus isolate 35/74 was used as the challenge virus. The virus was amplified and titrated on baby hamster kidney (BHK) cells cultured with CO_2_-independent medium (CIM, Invitrogen), supplemented with 5% FBS and 1% penicillin–streptomycin (Invitrogen). Titers were expressed as tissue culture infective dose 50 (TCID_50_) according the Spearman–Kärber algorithm (Spearman, 1908; Kärber, 1931). The virus was handled under biosafety level-3 laboratory conditions in class-III biosafety cabinets.

### Experiment A

**FIGURE 1:**
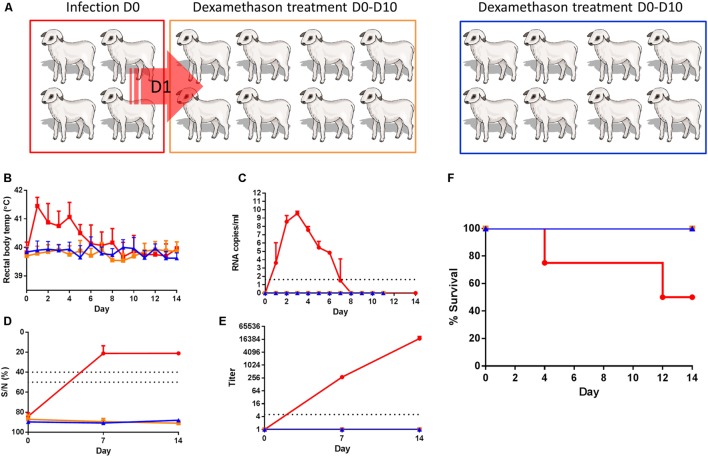
**Dexamethasone-treated lambs do not show any evidence of infection upon contact with RVFV viremic lambs. (A)** Cartoon representing the experimental design. Four lambs (red) were inoculated with RVFV via intravenous route and co-housed with eight dexamethasone-treated lambs (orange) the next day until the end of the experiment. Another group of 8 lambs functioned as a control group (blue). Rectal temperatures **(B)**, viremia **(C)**, antibody responses by ELISA **(D)**, and virus neutralization test (VNT) **(E)** and % survival **(F)** are depicted. Error bars represent averages with SD.

Twenty conventional European breed lambs, between 7 and 9 weeks of age, purchased from a commercial sheep farm in The Netherlands, were divided over one group of four animals and two groups of eight animals. Lambs were housed in biosafety level-3 containment facilities. Groups were color-coded according to their treatment (**Figure 1A**). On day 0 lambs from the red group (four animals) were sedated by intramuscular administration of 40 μg/kg medetomidine (Sedator^®^, Eurovet, The Netherlands) and subsequently inoculated with 1 ml cell culture medium containing 10^5^ TCID_50_ of RVFV strain 35/74 in the *vena jugularis* using a 18 gauge, 25 mm needle. The group of four animals was housed indoors for 1 day in a pen of 8 m^2^. The next morning, these inoculated lambs were brought into contact with eight sentinel lambs (orange group, **Figure 1A**). From this point onward, the lambs were held in a pen of 12 m^2^. From day 0 until day 10, the sentinel lambs were treated daily, via intramuscular injection, with 1 ml of 2 mg/ml dexamethasone (Pro Inj, Alfasan, Woerden, The Netherlands). A group of eight separately housed control lambs was treated with dexamethasone only (blue lambs, **Figure 1A**). Lambs were subsequently monitored for 14 days and EDTA blood samples for detection of viremia were collected daily up to day 12. Serum samples for analyses of antibody responses were collected weekly. To prevent possible predisposition to horizontal transmission by damaging mucosal tissues, no rectal or oral swabs were collected. At the end of the experiment (day 14) surviving lambs were euthanized by intravenous administration of 50 mg/kg sodium pentobarbital (Euthasol^®^, ASTfarma, The Netherlands) and subsequent exsanguination. Upon necropsy of contact and control animals, liver, spleen, lung and tonsils samples were collected. To prevent the risk of transmission of RVFV via aerosols, no necropsy was performed on lambs from the red group that succumbed to the infection.

### Experiment B

**FIGURE 2:**
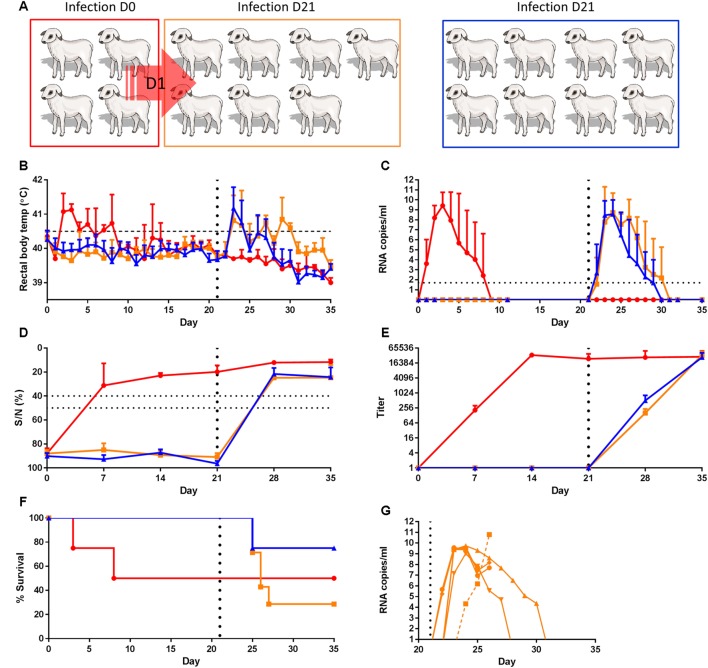
**Contact-exposure with viremic lambs does not affect the susceptibility to infection of healthy contact lambs. (A)** Cartoon representing the experimental design. Four lambs (red) were inoculated on day 0 (D0) with RVFV via intravenous route and co-housed with eight untreated lambs (orange) the next day. One lamb died from unrelated illness and was removed from the study. A group of eight lambs functioned as a control group (blue). On D21 contact-exposed lambs (orange) and control lambs (blue) were experimentally infected. Rectal temperatures **(B)**, viremia **(C)**, antibody responses by ELISA **(D)** and VNT **(E)** and % survival **(F)** are depicted. Error bars represent averages with SD. **(G)** displays viral RNA copies of each individual lamb of the orange group as determined from day 20, of which the average viral RNA levels are displayed in **(C)**. Measurements of samples of lamb 8836 are connected by a dashed line.

Experiment B was performed in parallel with experiment A, with another 20 lambs of the same age and supplier. Lambs were housed in pens as described above. These lambs were also divided over three groups consisting of one group of 4 (red group, **Figure 2A**) and two groups of 8 lambs (orange and blue, **Figure 2A**). The red group was treated similar as the red group of experiment A. The orange (contact-exposed) and blue (control) groups did not received dexamethasone treatment but were challenged with wild-type virus (1 ml, 10^5^ TCID_50_, strain 35/74) three weeks after contact exposure. Starting on day 21, EDTA blood samples for the detection of viremia and PAXgene blood samples (PreAnalytiX) for the analyses of cytokine responses, were collected daily. Serum samples for the detection of antibodies were collected weekly.

### Diagnostic Procedures

Detection of viral RNA was performed by quantitative real-time PCR (qRT-PCR) as described (Kortekaas et al., 2012). Briefly, RNA was isolated from plasma samples using the RNAeasy kit (Qiagen). Primers, probes and cycling conditions on the LightCycler were used as described previously (Kortekaas et al., 2012). Virus isolations from plasma samples were performed by incubation of 1:1 diluted samples with BHK cells. Inocula were replaced after 1.5 h incubation, and replaced with fresh culture medium. Five days post inoculation, cytopathic effect was scored.

Tissue homogenates for qRT-PCR analysis and virus isolation were prepared as described previously with some modifications (Kortekaas et al., 2012). Briefly, tissue homogenates for virus isolation were prepared by homogenizing approximately 1.5 g of tissue in a IKA^®^ Ultra Turrax tube DT20 (IKA) in 15 ml CO_2_-independent medium (Gibco). Cell debris was subsequently removed by slow-speed centrifugation and 200 μl was used for RNA isolation as described for the plasma samples. Cleared lysates (0.75 ml) were also directly added to BHK cells to achieve optimal sensitivity of virus isolation, in six well plates, and incubated for 2 h at RT. After replacement by fresh medium, the plates were incubated for 6 days at 37°C.

The commercial IDScreen^®^ Rift Valley Fever Competition ELISA (ID-VET, Montpellier, France) was used to detect antibodies against the RVFV N protein.

Virus neutralizing antibodies were detected using a recently developed highly sensitive virus neutralization test (VNT), which makes use of a 4-segmented RVFV expressing enhanced GFP (Wichgers Schreur et al., 2014, 2015). Briefly, ∼200 TCID_50_ of the RVFV-LMMS_eGFP_ virus was incubated with threefold serial dilutions of sera in 96-well plates. After a 2-h incubation period, 40,000 BHK cells were added per well. After 2 days incubation at 37°C and 5% CO_2_, eGFP expression was evaluated with an EVOS-FL microscope. VNT titers were calculated using the Spearman–Kärber algorithm.

### Cytokine mRNA Levels

RNA was isolated from peripheral blood mononuclear cells (PBMC) collected in PAXgene Blood RNA Tubes (PreAnalytiX). Briefly, PBMCs were pelleted and washed according the PAXgene protocol and lysed in 300 μl Trizol-LS (Invitrogen). Total RNA was subsequently extracted using the Direct-zol^TM^ RNA MiniPrep kit (Zymo research) according the manufacturers’ instructions. 50–200 ng RNA of each sample was subsequently reverse-transcribed using Oligo-Dt and Superscript III reverse transcriptase (Promega). SYBR green based qRT-PCRs of IL-8, IL-1β, IL-6, IL-10, TNF, and IFN-γ were performed as described previously (Wichgers Schreur et al., 2011). Primer sequences are listed in Supplemental Table 1. Gene expression levels were normalized with the house keeping gene *gapdh*. To determine gene-expression kinetics, fold-inductions compared to day 0 were calculated.

### Histopathology and Immunohistochemistry

Tissues for histopathology were fixed in 10% phosphate-buffered formalin for a minimum of 48 h and processed into paraffin. Sections of 4 μm were placed on silane-coated glass slides and dried for at least 48 h at 37°C. After deparaffinization and rehydration in graded alcohols, endogenous peroxidase activity was blocked in methanol/H_2_O_2_. Sections were pre-treated by 15 min autoclaving at 121°C in citrate buffer (pH 6.0). After cooling down, sections were incubated for 60 min with the monoclonal antibody 4-D4, which is directed against the Gn protein of RVFV (kindly provided by Dr. Connie Schmaljohn, USAMRIID). Mouse Envision horseradish peroxidase (Dakopatts, Denmark) was used as the secondary antibody and diaminobenzidine as the substrate.

## Results

### Contact Exposure of Dexamethasone-Treated Lambs (Experiment A)

To study if exposure to viremic lambs results in disease in immunocompromised lambs, 4 lambs were experimentally infected with RVFV via the intravenous route (**Figure 1A**, red group) and brought into contact with eight dexamethasone-treated sentinel lambs (**Figure 1A**, orange group) the day after inoculation. The lambs we co-housed for a period of 2 weeks. A control dexamethasone-treated group, consisting of another eight lambs was housed separately and was not brought into contact with RVFV-infected lambs (**Figure 1A**, blue group). The four inoculated lambs developed high fever (>41°C) which started on the day after challenge and lasted for several days (**Figure 1B**). Fever was associated with viremia as determined by qRT-PCR (**Figure 1C**). Earlier experiments with lambs have demonstrated that virus can be isolated from samples that contain viral RNA levels >10^5^ copies/ml as determined by the qRT-PCR employed in the present work (Kortekaas et al., 2012). In line with these earlier results, virus was isolated from all plasma samples with viral RNA levels >10^5^ copies/ml (data not shown). One lamb succumbed to the infection on day 4 and one on day 12 (**Figure 1F**). None of the dexamethasone-treated lambs developed fever (>40.5°C) or viremia, and none of these lambs developed any sign of disease that could be associated with horizontally transmitted RVFV. Although RVFV-specific antibodies were detected by ELISA and VNT in the sera of the two surviving lambs of Group 1A (**Figures 1D,E**), none of the dexamethasone-treated lambs developed RVFV-specific antibodies. After day 14, all surviving lambs were euthanized and samples of the liver, spleen, lung and tonsils were collected. From the organ samples collected from the two lambs of the red group that recovered from disease, virus was isolated from tonsils. In addition, virus was isolated from the spleen of one lamb (data not shown). In the samples collected from the contact-exposed, dexamethasone-treated lambs, neither infectious virus nor viral RNA were detected.

### Contact Exposure and Subsequent Challenge Infection of Lambs (Experiment B)

As an additional hypothesis, we considered the possibility that horizontally transmitted RVFV to immunocompetent animals changes the susceptibility to mosquito mediated infection. To test this hypothesis, a group of four lambs was infected via the intravenous route (**Figure 2A**, red group) and brought into contact with eight healthy lambs (**Figure 2A**, orange group). The lambs we co-housed for a period of three weeks. Another group of eight lambs, housed separately, functioned as a control group (**Figure 2A**, blue group). All four experimentally infected lambs displayed fever associated with viremia by day 2 (**Figure 2B**). One lamb died on day 3 and one on day 8 (**Figure 2F**). Apart from the lamb that died on day 3, antibodies were detected by both ELISA and VNT in the remaining three lambs (**Figures 2D,E**). No clinical signs were observed in contact-exposed lambs and no viral RNA or RVFV-specific antibodies were detected in these animals until day 21 (**Figure 2**). Of note, one of the contact-exposed lambs died on day 17 from peritonitis resulting from purulent omphalophlebitis and was removed from the study.

**FIGURE 3:**
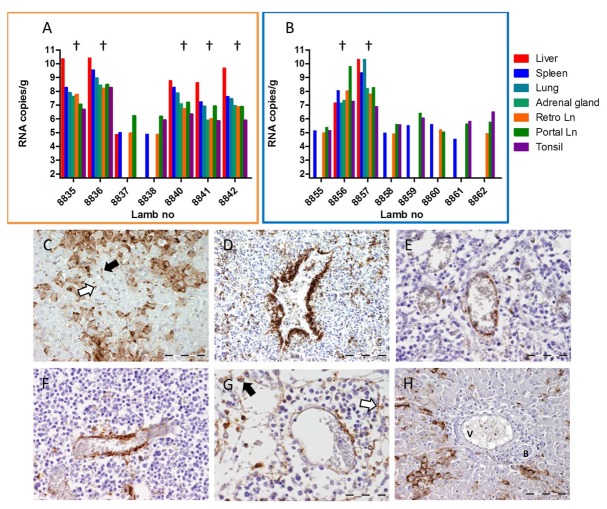
**Detection of viral RNA and viral antigen in organ samples collected from animals of Experiment B.** Detection of viral RNA by qRT-PCR in organ samples collected from contact-exposed and subsequently challenged lambs (orange frame, **A**) and of infected control lambs (blue frame, **B**). Lambs that succumbed to the infection are marked (†). **(C–H)** Immunohistochemical staining of organ samples collected from lamb 8836. **(C)** Detail of liver acinus with strong immunostaining of hepatocytes. Also note the accumulation of RVFV antigen in Kupffer cells (black arrow) and circulating macrophages (white arrow) within the liver sinusoids. **(D–G)** Positive staining for RVFV antigen in the endothelial cells of venules and veins in **(D)** spleen; **(E)** tonsil; **(F)** paracortex of the retropharyngeal lymph node; and **(G)** medulla of the portal lymph node. Also note the positive staining in the circulating macrophages (black arrow) and littoral cells (white arrow) in the medullary sinuses of the lymph node. **(H)** Portal area of the liver. Note the heavy staining for RVFV antigen in the hepatocytes and the absence of staining of the endothelial layer of the Vena porta (V). B = bile duct. Bar = 100 μm **(C,D,H)** and 50 μm **(E,F,G)**.

On day 21, contact-exposed lambs and control lambs were inoculated with RVFV. All lambs from both groups developed fever (**Figure 2B**) and viremia (**Figure 2C**) within two days after inoculation. All surviving lambs developed antibodies as determined by ELISA (**Figure 2D**) and VNT (**Figure 2E**). The percentage of mortality among experimentally infected lambs was 25% (**Figure 2F**), which is in line with results from several previous studies with lambs of 11–13 weeks of age. Remarkably, mortality in challenged, contact-exposed lambs was 71%, which is exceptionally high. This high percentage of fatality was associated with a higher average level of viremia and prolonged fever in all lambs, although the differences between the groups were not statistically significant. RVFV RNA levels in liver, spleen, adrenal gland, lung, retropharyngeal lymph nodes, portal lymph nodes, and tonsil samples of these animals were also very high (**Figures 3A,B**). Similar as previously observed, the majority of animals that died shortly after the challenge with RVFV revealed an extensive necrosis of the liver with bridging of necrotic areas between neighboring liver acini. Immunohistochemistry staining showed a massive presence of RVF antigen within the degenerating and necrotic hepatocytes (data not shown). In addition, RVFV antigen was detected in the mononuclear phagocyte system of the liver (**Figure 3C**), spleen, and lymph nodes as reported previously. Interestingly, an unusual pattern was observed in the immunohistochemistry analysis of lamb with number 8836. This lamb developed a delayed, but extremely high viremia, approaching 10^11^ copies/ml (dashed line in **Figure 2G**). Of note, also the titer of infectious virus reached the highest level in this animal (>6.3 TCID_50_/ml). Immunohistochemistry revealed a heavy staining of the endothelial cells lining both smaller and larger veins in the spleen, tonsil, and lymph nodes (**Figures 3D–G**). Remarkably, staining of endothelial cells was confined to lymphoid organs, as endothelial cells in the liver and adrenal gland were negative for RVFV antigen (**Figure 3H**).

**FIGURE 4:**
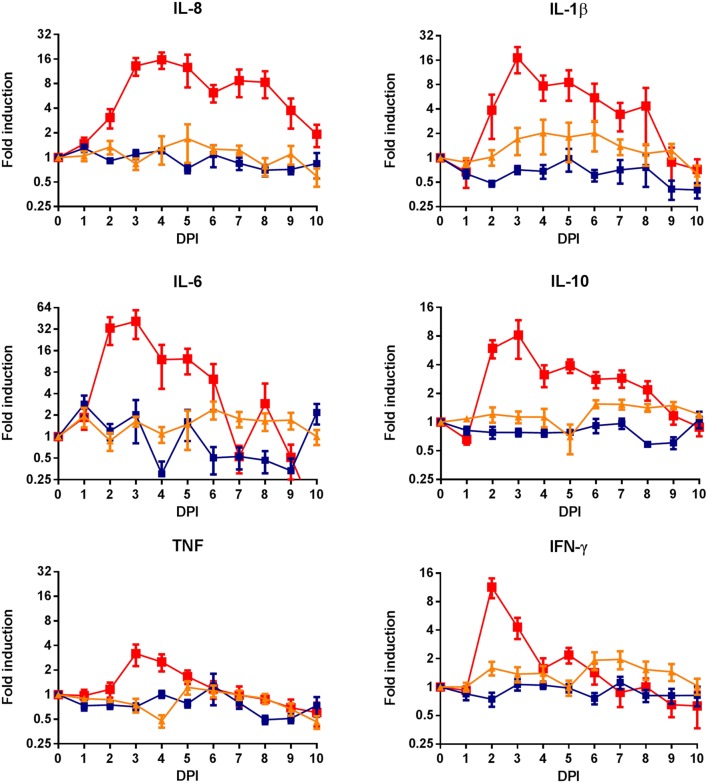
**Cytokine mRNA responses in lambs following RVFV infection or contact-exposure.** Whole blood samples were collected from lambs experimentally infected with RVFV (red), contact-exposed lambs (orange) and separately housed, non-exposed control lambs (blue). Blood samples were collected in PAXgene tubes for a period of 10 days. RNA was isolated from PBMCs and used for reverse-transcription. Results represent SYBR green based qRT-PCRs of IL-8, IL-1β, IL-6, IL-10, TNF-α, and IFN-γ. Gene expression levels were normalized with the house keeping gene *gapdh*. To determine gene-expression kinetics, fold-inductions compared to day 0 were calculated. Error bars represent averages with SEM.

Although contact-exposure did not result in disease, viremia or antibody responses, we anticipated the possibility that horizontally transmitted virus may induce subclinical infections and associated innate immune responses. Systemic innate immune responses were monitored by measuring cytokine mRNA levels for a period of 10 days. The results show that experimental RVFV infection results in strong pro-inflammatory cytokine responses. However, no significant differences in cytokine mRNA levels between contact-exposed lambs and control lambs were observed (**Figure 4**).

## Discussion

Rift Valley fever virus causes high viremia in domesticated ruminants, but is not present in appreciable amounts in urine, faces, or milk (Daubney et al., 1931; Easterday, 1965). The virus can, however, be detected by PCR or be isolated from oral or rectal swabs of infected ruminants (Saber et al., 1984; Busquets et al., 2010, 2014) and was also isolated from pharyngeal or throat washings from humans (Francis and Magill, 1935; Abdel-Wahab et al., 1978; Imam et al., 1979). Considering these findings and the high infectivity of the virus when delivered via oral route or respiratory route (Hartman et al., 2013; Reed et al., 2013) it seems contradictory that horizontal transmission rarely occurs in nature.

Harrington et al. (1980) were the first to report horizontal transmission among sheep under experimental conditions. One of four contact control sheep developed clinical disease and detectable viremia at 7 days post exposure (Harrington et al., 1980). The transmission route remained unknown, as the sheep had not been in contact with ewes that aborted or with sheep that had detectable virus in their saliva. More recently, Busquets and co-workers described transmission of the virus from lamb to lamb, and also in this case, the transmission route remained unclear (Busquets et al., 2010).

When we designed the present study, we hypothesized that ruminants may be more susceptible to horizontally transmitted RVFV during periods of immunosuppression. Immunosuppression can result from co-infections or from stressful conditions. Naturally occurring stressful conditions may lead to increases in endogenous corticosteroids, which are potent immunosuppressive agents. In the current work, the effect of increased endogenous corticosteroids was simulated by treatment with the synthetic glucocorticosteroidsteroid dexamethasone, which was previously successfully used in sheep to re-activate bovine herpesvirus type-5 (Silva et al., 1999). Exposure of eight dexamethasone-treated lambs to four highly viremic lambs, of which two succumbed to the infection, did not result in signs of disease that could be attributed to RVFV infection and no viral RNA was detected or viable virus isolated from plasma samples. Despite optimized virus isolation procedures, no virus was isolated from liver, spleen, lung and tonsil samples collected from dexamethasone-treated contact lambs. From this, we conclude that either no horizontal transmission occurred in this experiment, or that horizontal transmission did not result in productive infection.

As a second hypothesis, we considered the possibility that horizontally transmitted virus induces an immune response that could change the susceptibility to infection via mosquito bite. This possibility was considered, as in previous vaccination/challenge studies with lambs, we occasionally found animals that were protected from disease in the apparent absence of vaccine-induced neutralizing antibodies (unpublished observations). Apart from low levels of antibodies, this protection could also be mediated by innate or cellular immune responses, which we intended to detect indirectly, by measuring cytokine responses. Considering that no antibody or cytokine responses were detected in contact-exposed lambs and the fact that these lambs were equally susceptible to subsequent challenge infection, we conclude that either no virus was horizontally transmitted in this experiment, or that horizontal transmission of RVFV does not result in protective immune responses.

A striking finding in our study involved one of the contact-exposed and subsequently challenged lambs. This lamb revealed signs of infection 1–2 days later than usual and subsequently developed extremely high viremia approaching 10^11^ copies/ml within three days post inoculation (dashed line in **Figure 2G**). The lamb succumbed to the infection 3 days after onset of viremia and was found to have significantly higher viral loads in organs. Remarkably, this seemingly delayed onset, but subsequently fast development of viremia, disease and mortality was associated with detection of viral antigen in endothelial cells of lymphoid organs. Although this phenomenon may have been caused by a rare event in the host, the possibility should be considered that one or several mutations in the RVFV genome were responsible for this finding. If this is indeed the case, this RVFV variant might have evolutionary advantage over the remaining population, considering the unusually high viremia levels that were detected. Clearly, this intriguing finding warrants further study.

## Conclusion

Our results provide no evidence that horizontal transmission of RVFV occurred in the present experiments. Considering the previous findings of Harrington et al. (1980) and Busquets et al. (2010) the possibility must be considered that damage of mucosal tissues as a result of swabbing could have increased both the shedding potential of viremic animals as well as the susceptibility of contact-exposed animals in these former studies. In this light, it must be considered that co-infections that damage mucosal surfaces may predispose for horizontal transmission of RVFV in the field.

## Author Contributions

PS, LvK, NO, RM, JK: Designed the experiment and interpreted results. PS, JK: Wrote the manuscript. PS, LvK, NO, JA: Performed the experiments.

## Conflict of Interest Statement

The authors declare that the research was conducted in the absence of any commercial or financial relationships that could be construed as a potential conflict of interest.
