# Supplementary material for: Co-housing of Rift Valley Fever Virus Infected Lambs with Immunocompetent or Immunosuppressed Lambs Does Not Result in Virus Transmission
Source: Front Microbiol. 2016 Mar 7;7:287. doi: 10.3389/fmicb.2016.00287 (PMC4779905; doi:10.3389/fmicb.2016.00287)
Supplement: Supplementary file 1 [file Table_1.DOCX]

**Supplemental Table 1. Primers used for qPCR of cytokine mRNA**

| Name | Sequence | Type^a^ |
| --- | --- | --- |
| JR746 | GAATGACCTGTCGCCAAAATC | INF-γ (F) |
| JR747 | TTGCAGGCAGGAGAACCATTA | INF-γ (R) |
| JR750 | GGTCCACGTGGGCTGAATAA | IL 1b (F) |
| JR751 | GCAGTTGGGCATGGATCAC | IL 1b (R) |
| JR756 | CCTTGAGAATTTCCTGCAGTTCA | IL 6 (F) |
| JR757 | CTGACCAGAGGAGGGAATGC | IL 6 (R) |
| JR758 | AATCCTTTTTCCATTGCTTCCA | IL 8 (F) |
| JR759 | TACACCAGACCCACACAGTACTCA | IL 8 (R) |
| JR760 | TGACTGCCCTCTAATTTCTCTTGTC | IL 10 (F) |
| JR761 | GGCTCCCTGGTTTCTCTTCCT | IL 10 (R) |
| JR762 | TTTGTGCCTCCTTTTGCTTATG | TNF (F) |
| JR763 | TTCAGTGATGTAGCGACAAATCAG | TNF (R) |
| JR764 | CACTCACTCTTCTACCTTCGATGCT | GAPDH (F) |
| JR765 | GTTGCTGTAGCCGAATTCATTG | GAPDH (R) |

^a^(F); Forward primer, (R); Reverse primer
